# Cryptotanshinone Ameliorates Radiation-Induced Lung Injury in Rats

**DOI:** 10.1155/2019/1908416

**Published:** 2019-02-20

**Authors:** Yifang Jiang, Fengming You, Jie Zhu, Chuan Zheng, Ran Yan, Jinhao Zeng

**Affiliations:** ^1^School of Basic Medical Sciences, Chengdu University of Traditional Chinese Medicine, Chengdu 610075, China; ^2^School of Clinical Medicine, Chengdu University of Traditional Chinese Medicine, Chengdu 610075, China; ^3^Cancer Institute, Chengdu University of Traditional Chinese Medicine, Chengdu 610075, China

## Abstract

Cryptotanshinone (CTS) was reported to repress a variety of systemic inflammation and alleviate cardiac fibrosis, but it is still unclear whether CTS could prevent radiation-induced lung injury (RILI). Here, we investigated the effects and underlying mechanisms of CTS on a RILI rat model. Our data revealed that CTS could efficiently preserve pulmonary function in RILI rats and reduce early pulmonary inflammation infiltration elicited, along with marked decreased levels of IL-6 and IL-10. Moreover, we found that CTS is superior to prednisone in attenuating collagen deposition and pulmonary fibrosis, in parallel with a marked drop of HYP (a collagen indicator) and *α*-SMA (a myofibroblast marker). Mechanistically, CTS inhibited profibrotic signals TGF-*β*1 and NOX-4 expressions, while enhancing the levels of antifibrotic enzyme MMP-1 in lung tissues. It is noteworthy that CTS treatment, in consistent with trichrome staining analysis, exhibited a clear advantage over PND in enhancing MMP-1 levels. However, CTS exhibited little effect on CTGF activation and on COX-2 suppression. Finally, CTS treatment significantly mitigated the radiation-induced activation of CCL3 and its receptor CCR1. In summary, CTS treatment could attenuate RILI, especially pulmonary fibrosis, in rats. The regulation on production and release of inflammatory or fibrotic factors IL-6, IL-10, TGF-*β*1, NOX-4, and MMP-1, especially MMP-1 and inhibition on CCL3/CCR1 activation, may partly attribute to its attenuating RILI effect.

## 1. Introduction

Radiation-induced lung injury (RILI) is a common and fetal complication of thoracic radiotherapy. RILI develops in almost ~5% to 20% lung cancer patients receiving radiotherapy [1] and generally leads to discontinuation of treatment. RILI is a complex pathological process mainly comprising early pneumonitis and late pulmonary fibrosis. Firstly, radiation leads to early damage of epithelial cells and inflammatory response. Next, impaired cells provoke the production and release of various inflammatory cytokines, accompanying with macrophages or neutrophils infiltration in lung tissue. In parallel, to protect against cellular injury and death, impaired cells also induce the production and release of proliferative molecules or profibrotic cytokines such as transforming growth factor-*β* (TGF-*β*) [2], connective tissue growth factor (CTGF) [3], and alpha-smooth muscle actin (*α*-SMA) [4], leading to fibroblast replication and differentiation of fibroblast into myofibroblast. Finally, myofibroblasts produce an abundance of collagen sedimentation leading to lung fibrosis. The progressive replacement of the normal lung interstitium by fibrous tissues results in restriction of lung and gradual loss of function [5].

Macrophage inflammatory protein 1-alpha (CCL3) and its receptor CC chemokine receptor 1 (CCR1) mediate production and secretion of several key growth factors and cytokines as well as chemokine mediators [6–8], thereby playing a key role in the regulation of both inflammatory response and fibrotic process. Chemokine CCL3 dose dependently inhibited ATP-stimulated release of interleukin- (IL-) 1*β* by monocytic cells [9]. CCR1-FcepsilonRI costimulation significantly enhanced secretion of transforming growth factor beta-1, tumor necrosis factor-alpha, and the cytokine IL-6 in mast cells [7]. In return, interleukins secretion affects CCL3/CCR1-associated pathologic processes, as evidenced by a report which revealed that IL-10 neutralization in Streptococcus pneumoniae infected mice was associated with augmented neutrophil recruitment and resulted in enhanced CCL3 production [10]. Also, CCR1 has been reported to regulate the recruitment of CD45(+) Collagen I (+) fibrocytes which is implicated in fibrogenic liver injury, as demonstrated by 25% inhibition of fibrocyte migration in CCR1-/- wt mice [11]. Moreover, CCL3 knockout mice displayed less inflammatory infiltrate in adenine-injured renal tissue, which also contained a striking reduction of collagen deposition and a less production of TNF-*α* [12]. The aforementioned studies revealed the contribution of CCL3/CCR1 activation in both inflammatory response and fibrotic process, and CCL3/CCR1 might serve as potential targets to ameliorate RILI. However, few studies focus on CCL3/CCR1 linking to RILI.

Currently, RILI treatment often invokes general anti-inflammatory agent corticoid clinically; various adverse effects, however, limit its application. RILI patients receiving long corticoid intervention may have varying degrees of compromised immunity, osteoporosis, peptic ulcer, hypertension, water-sodium retention, hypopotassemia, glucose-lipid metabolic disorder, etc. Hence, there is a compelling need to find novel therapies to alleviate RILI. Cryptotanshinone (CTS) is a major lipophilic extraction from* Salvia miltiorrhiza Bunge* (Danshen), an herbal dietary supplement documented in the USP 37-NF32. Also, in China, two certified compound preparations “Tanshinones capsules” (Z13020110) and “Compound Danshen dripping pills” (Z10950111), of which CTS is the major component [13, 14], were widely used more than 15 years in the market. Numerous clinical studies suggested their benefits, with no adverse events, were found, in treating acne [15], otitis externa [16], ulcerative colitis [17], hepatitis B with liver fibrosis [18], coronary heart disease [19], and hypertension [20]. Previously, CTS has been proved to be a safe extraction [14] and possess antineoplastic [21, 22], antioxidative [23], and anti-inflammatory [24, 25] bioactivities. Recently, researchers revealed that CTS was also capable of alleviating cardiac fibrosis by depressing signal transducers and activators of transcription 3 (STAT3) [26], Cyclooxygenase-2 (COX-2), NADPH oxidase-2 (NOX-2), and NOX-4 signals [27] and upregulating matrix metalloproteinase-2 (MMP-2) activation [28]. In addition, CTS was found to inhibit profibrotic activities of hypertrophic scar fibroblasts and accelerates wound healing [29]. However, the potential therapeutic effect of CTS on radiation-induced pneumonitis and fibrosis remains untested.

In this study, using a RILI rat model, we first tested whether CTS ameliorate early pneumonitis and late pulmonary fibrosis. In parallel, we observed the regulatory effects of CTS on several key inflammatory cytokines and on multiple profibrotic or antifibrotic factors. Finally, we investigated whether its potential therapeutic effect on RILI was contributed by inhibition of CCL3/CCR1 activation.

## 2. Materials and Methods

All procedures in this study were approved by the Institutional Animal Care and Use Committee (IACUC), Chengdu University of Traditional Chinese Medicine, and performed in accordance with the approved guidelines set forth by IACUC.

### 2.1. Animals

Ninety-six male Sprague-Dawley rats (8 weeks of age, weighing 180-220g) were obtained from Chengdu Dashuo Experimental Animal Co., Ltd. (SCXK-2013-24). Animals were housed under controlled conditions of 22±2°C and 60-80% relative humidity and fed with standard rodent chow and water.

### 2.2. Drugs

Cryptotanshinone (purity surpass 95%) was purchased from Xi'an He Lin Biological Engineering Co., Ltd. (110852-200806, Xi'an, China). Prednisone was obtained from Shanghai Xinyi Pharmaceutical Co., Ltd. (H31020675, Shanghai, China).

### 2.3. RILI Rat Model and Treatments

After acclimatizing with the facilities for one week, the animals were randomly assigned to the following four groups: (i) normal rats, n=24; (ii) radiation (RT) rats, n=24, the rats received lung radiation; (iii) RT+ Prednisone (PND): the rats received lung radiation plus 3.12 mg/kg PND oral administration, n=24; (iv) RT+ CTS: the rats received lung radiation plus 20 mg/kg CTS oral administration, n=24. Radiated rats were prescribed with whole lung radiation at a single dose of 14 Gy (6MV X-rays; 200kVp) after anesthetizing with intraperitoneal injection of 0.3 mL/100g chloral hydrate. During the radiation, rats were shielded with leads to protect their head, abdomen, and extremities. On the second day after radiation, treated rats were given PND or CTS by gastrogavage once a day and five days a week. Six rats in each group were sacrificed with anesthetization at months 1, 3, 5, and 8 after treatment.

### 2.4. Pulmonary Function Testing

Tidal volume, maximal voluntary ventilation, and respiratory rate were measured with an unrestrained whole body plethysmograph (Buxco, Wilmington, NC, USA) at months 1, 3, 5, and 8.

### 2.5. Pulmonary Coefficient

After sacrificing the rats, the lungs were removed, trimmed of extraneous tissue. Pulmonary coefficient was calculated by the following equation: lung wet weight (g) / body weight (kg) × 100%.

### 2.6. Pathological Analysis

Lung specimens were fixed in 10% neutral-buffered formalin overnight, embedded in paraffin, and then sliced into 5 *μ*m sections. The sections were stained with hematoxylin and eosin (H&E) and Masson's trichrome. The results of H&E staining were graded according to inflammatory infiltration, interstitial edema, alveolar wall integrity, and septal wall thickness. The grading was scored as follows: score 0, normal; score 1, mild (appearance of some inflammatory cells and mild focal edema, slightly widened alveolar septa); score 2, moderate (relatively apparent inflammatory infiltration, moderate edematous swelling and septal wall thickening, occasional discontinuity in the wall of alveolus); score 3, severe (numerous inflammatory cells infiltrated in alveolar septae that is severely thickened, considerable alveolar flooding, with heavy deposition of collagen, frequently occurrence of discontinuity of alveolar wall). The Masson's trichrome staining was performed to determine the degree of interstitial fibrosis. Collagen sedimentation was assessed visually by range and distribution of blue-stained tissues. Criteria for scoring were as follows: score 0, normal; score 1, mild (slight fibrous thickening of alveolar or bronchiolar walls, without obvious lung architecture impairment); score 2, moderate (increased fibrosis with definite damage to lung architecture, and formation of small fibrous masses); score 3, severe (large fibrous areas and severe distortion of lung architecture; “honeycomb lung” could be found). Three random visual fields were selected from a slice, and mean score of the slice was taken. These sections were evaluated by two independent pathologists in a blinded manner.

### 2.7. Enzyme-Linked Immunosorbent Assay

The concentrations of IL-6, IL-10, CTGF, and HYP were measured using ELISA Kit (Boster Biological Engineering Co., Ltd., Wuhan, China), according to the manufacturer's instructions. The optical density (OD) value was determined at 450 nm using ELISA reader (Bio-Tek Instruments Inc., Winooski, VT, USA).

### 2.8. Immunohistochemistry Analysis

We preformed routine immunohistochemistry to measure the expression of *α*-SMA, TGF-*β*1, CTGF, NOX-4, NOX-2, COX-2, STAT3, MMP-1, MMP-2, MMP-3, MMP-7, MMP-9, CCL3, and CCR1 in lung tissues. Briefly, sections were incubated with antibodies against *α*-SMA (1:1000, ab124964, Abcam, Cambridge, UK), TGF-*β*1 (1:100, MAB240, R&D system, Minneapolis, USA), CTGF (1:200, ab6992, Abcam, Cambridge, UK), NOX-4 (1:150, ab109225, Abcam, Cambridge, UK), NOX-2 (1:500, ab31092, Abcam, Cambridge, UK), COX-2 (1:100, ab52237, Abcam, Cambridge, UK), STAT3 (1:75, ab76315, Abcam, Cambridge, UK), MMP-1 (1:200, ab137332, Abcam, Cambridge, UK), MMP-2 (1:125, ab37150, Abcam, Cambridge, UK), MMP-3 (1:50, ab52915, Abcam, Cambridge, UK), MMP-7 (1:75, ab5706, Abcam, Cambridge, UK), MMP-9 (1:250, ab76003, Abcam, Cambridge, UK), CCL3 (0029590101, ABclonal, China), and CCR1 (ZP4113BBP13, Boster, China) at 4°C overnight. After washing off the unbound antibodies with PBS, the sections were incubated with biotinylated anti-rabbit secondary antibody (ZSGB-BIO, Beijing, China) at room temperature for 1 h and then were visualized with diaminobenzidine. Intensity and quantity of stained sections was evaluated using a semiquantitative immunoreactivity score (IRS) [30]. Three random visual fields were selected from a section to score staining intensity (scored as A) and positive cell ratio (scored as B), that is, for A: 0, negative staining; 1, light-yellow staining; 2, pale-brown staining; 3, dark-brown staining. In parallel, for B: 0, no cells stained; 1, fewer than 10% of cells stained; 2, 10% to 50% of cells stained; 3, 51% to 80% of cells stained; 4, more than 81% of cells stained. The average of five fields was the final score of each section (calculated by multiplying A and B).

### 2.9. Statistical Analysis

Data were expressed as mean ± SEM. Multiple comparison was assessed by one-way analysis of variance (ANOVA) using SPSS 17.0 software.* P* <0.05 was considered to be significant.

## 3. Results

### 3.1. CTS Prevented Weight Loss of RILI Rats

**Figure 1:**
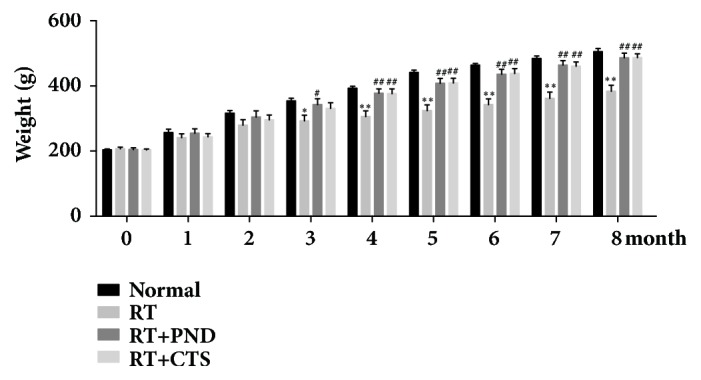
CTS prevented weight loss of RILI rats.* n* =6. ^*∗*^*P*< 0.05 and ^*∗∗*^*P* < 0.01 versus Normal; ^#^*P* < 0.05 and ^##^*P* < 0.01 versus RT.

First, we observed the impacts of CTS on the body weight, which serve as an important indicator of the general health condition, in RILI rats. Model RILI rats had notable lower body weight after month 3, as compared to control rats (Figure 1). CTS administration could partly preserve body weight, suggesting the rescuing efficacy of CTS in radiation-induced lung injury.

### 3.2. CTS Preserved Pulmonary Function of RILI Rats

**Figure 2:**
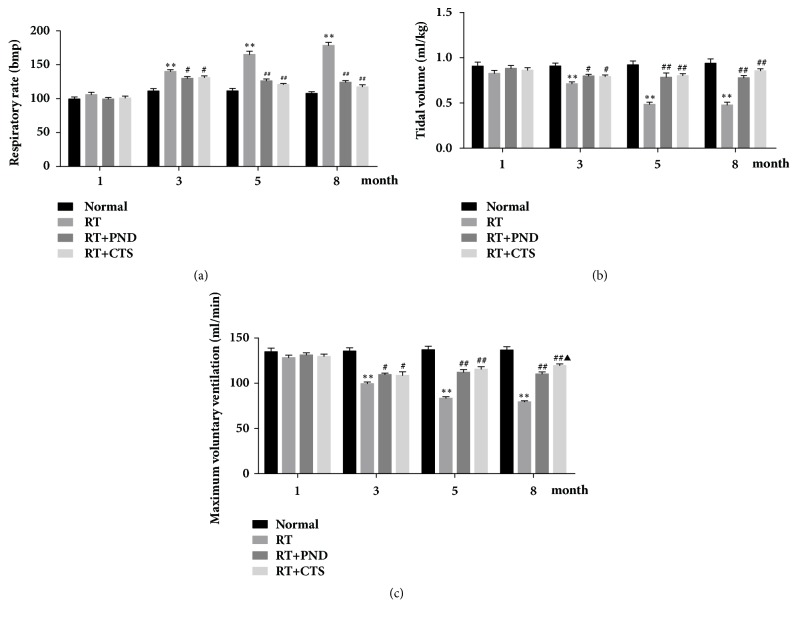
Effects of CTS on pulmonary function of RILI rats. (a) Respiratory rates. (b) Tidal volume. (c) Maximum voluntary ventilation.* n* = 6. ^*∗*^*P*< 0.05 and ^*∗∗*^*P* < 0.01 versus Normal; ^#^*P* < 0.05 and ^##^*P* < 0.01 versus RT; ^▲^*P*< 0.05 and ^▲▲^*P*< 0.01 versus RT+PND.

Tidal volume, maximum voluntary ventilation, and respiratory rates were evaluated as crucial indicators in pulmonary function testing. As compared with controls, model RILI rats had marked lower tidal volumes and maximum voluntary ventilation, as well as higher respiratory rates after 3-month radiation, which suggest a decline of pulmonary function seen in RILI rats. CTS treatment was able to partly restore the tidal volume, maximum voluntary ventilation, and respiratory rates (Figure 2). Our observation indicated that CTS intervention could restore the pulmonary function of RILI rats.

### 3.3. CTS Ameliorated RILI Histopathology

**Figure 3:**
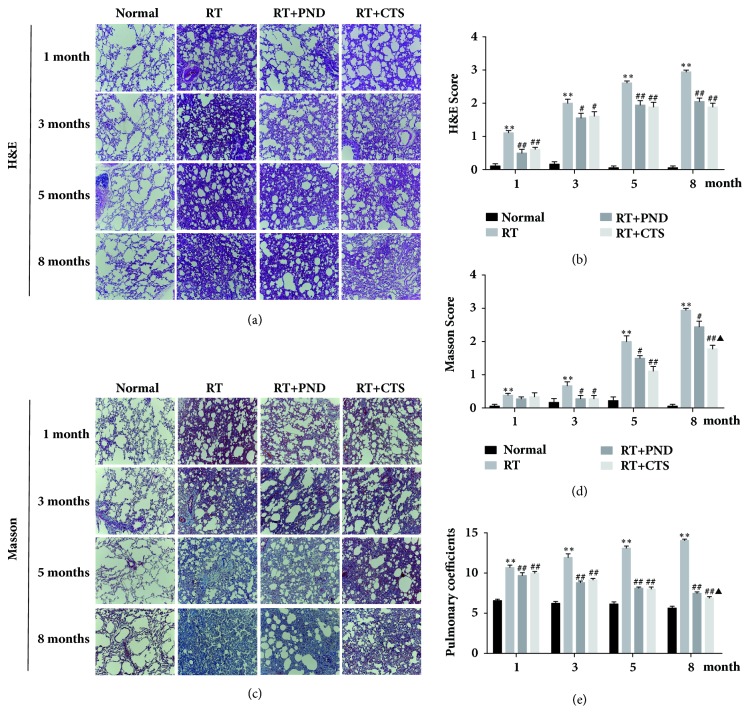
CTS ameliorated pulmonary coefficient and histopathology abnormalities in RILI rats. (a) H&E-stained lung tissue in each group (magnification × 20). (b) Semiquantitative analysis of H & E staining. (c) Masson's trichrome-stained lung tissue in each group (magnification × 20). Blue indicates the collagen deposition. (d) Semiquantitative analysis of Masson's trichrome staining. (e) Pulmonary coefficient of rats in each group.* n* = 6. ^*∗*^*P*< 0.05 and ^*∗∗*^*P* < 0.01 versus Normal; ^#^*P* < 0.05 and ^##^*P* < 0.01 versus RT; ^▲^*P*< 0.05 and ^▲▲^*P*< 0.01 versus RT+PND.

We further examined the pulmonary coefficient and pathological changes in rats from various groups. Pulmonary coefficient is a wet/weigh index of lung and reflects the degree of lung inflammation and fibrosis. As displayed in Figure 3(e), pulmonary coefficients were significantly increased in model RILI rats after 1-month radiation. Both CTS and PND treatments potently restored the pulmonary coefficients of RILI rats.

We also observed the pathological changes in lung tissues on months 1, 3, 5, and 8 by H&E staining; a visible difference between control and model rats was identified in terms of pathological alterations. Lung tissues from model RILI rats presented thickening of the alveolar septa, infiltration of inflammatory cells, and interstitial edema. Moreover, discontinuity in the wall of alveolus was observed after 5-month radiation. Both CTS and PND treatments markedly reduced the radiation-induced morphologic abnormalities (Figures 3(a) and 3(b)). At late phase of RILI, excessive deposition of collagen generally results in progressive loss of pulmonary function. Masson's trichrome staining depicted no noteworthy evidence of pulmonary fibrosis at early stage of radiation. After 5-month radiation, model RILI rats experienced a robust accumulation of collagenous fibers in thickened alveolar septa and the areas around the bronchiolar. Both CTS and PND treatments significantly attenuated the pulmonary fibrosis, which was reflected by the reduction of collagen sedimentation in lung tissues (Figures 3(c) and 3(d)). Interestingly, CTS exhibited stronger antifibrosis activity than PND treatment, especially after 5-month administration.

### 3.4. CTS Reduced *α*-SMA and HYP Levels in RILI Rats

**Figure 4:**
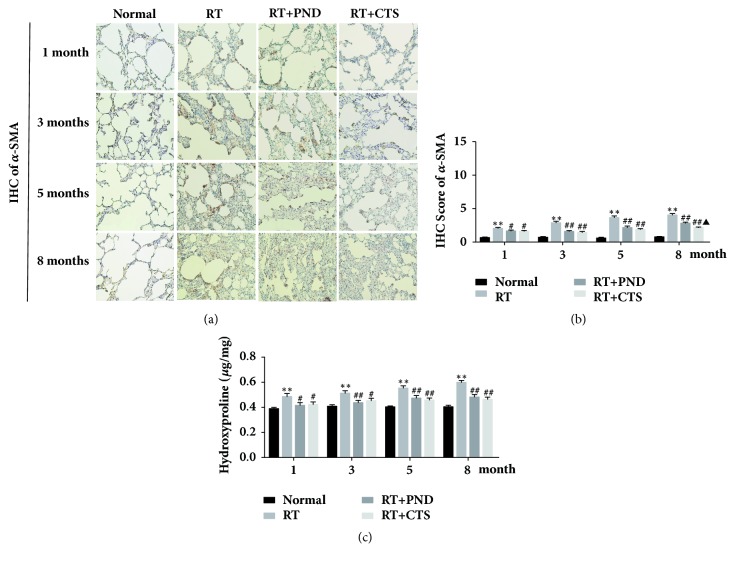
CTS reduced *α*-SMA and HYP levels in RILI rats. (a) IHC staining of *α*-SMA in lung tissue sections from each group (magnification × 200). (b) Semiquantitative analysis of *α*-SMA IHC staining. (c) HYP levels of lung homogenates in each group.* n* = 6. ^*∗*^*P*< 0.05 and ^*∗∗*^*P* < 0.01 versus Normal; ^#^*P* < 0.05 and ^##^*P* < 0.01 versus RT; ^▲^*P*< 0.05 and ^▲▲^*P*< 0.01 versus RT+PND.

*α*-SMA is considered as a specific marker of myofibroblasts, which plays a dominant role in fibrogenesis. Lung tissue immunostaining revealed that *α*-SMA levels were gradually and notably elevated with the increment of time after radiation, suggesting that fibrocytes gave rise to increased number of myofibroblasts in injured lung tissue. CTS treatment caused an astonishing reduction of *α*-SMA levels in lung tissues, and its inhibitory effect was proved to be significantly stronger than that of PND, especially at month 8 (Figures 4(a) and 4(b)).

HYP is a component of the protein collagen and thus plays a crucial role in collagen stability. We thus assayed HYP levels in lung tissue homogenates. As depicted in Figure 4(c), radiation led to significant increase of HYP content, which could be reversed remarkably by both CTS and PND treatments. These results indicated that CTS could suppress excessive activation of myofibroblasts, thereby reducing the collagen sedimentation and then attenuate radiation-induced pulmonary fibrosis.

### 3.5. CTS Decreased TGF-*β*1 Production in RILI Rats

**Figure 5:**
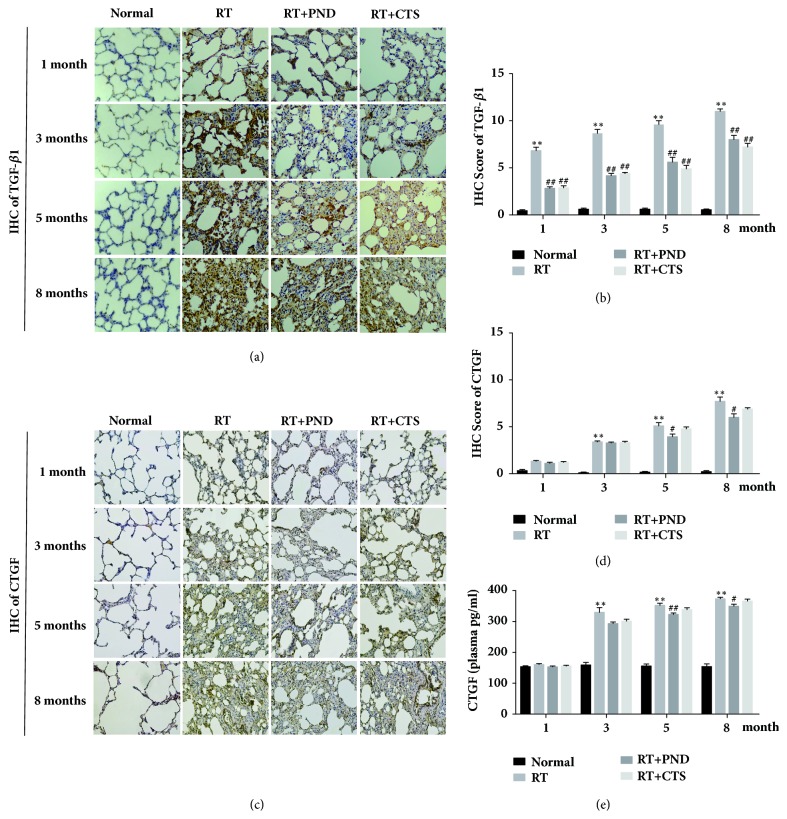
Effects of CTS on TGF-*β*1 and CTGF levels in RILI rats. (a) IHC staining of TGF-*β*1 in lung tissue sections from each group (magnification × 200). (b) Semiquantitative analysis of TGF-*β*1 IHC staining. (c) IHC staining of CTGF in lung tissue sections from each group (magnification × 200). (d) Semiquantitative analysis of CTGF IHC staining. (e) Plasma CTGF levels in each group.* n* = 6. ^*∗*^*P*< 0.05 and ^*∗∗*^*P* < 0.01 versus Normal; ^#^*P* < 0.05 and ^##^*P* < 0.01 versus RT.

To further elucidate the protective effect of CTS against radiation-induced pulmonary fibrosis, we measured the profibrotic cytokines TGF-*β*1 and CTGF in treated rats. TGF-*β*1 is a crucial profibrotic cytokine and has a huge role in initiating differentiation of fibroblasts into myofibroblasts and accelerating the synthesis of collagen, as evidenced by a large number of studies [31]. Lung tissue immunostaining displayed a gradual and significant increase of TGF-*β*1 expression in model RILI rats at months 1, 3, 5, and 8 after radiation, respectively. CTS and PND treatments could potently suppress TGF-*β*1 releasing, and we observed a slight stronger inhibition effect of CTS treatment when compared to PND treatment, although it did not achieve statistical significance (Figures 5(a) and 5(b)).

CTGF is a member of the Cyr61-CTGF-Nov protein family, and it is recognized to be implicated in fibroblast proliferation and collagen synthesis.  Figure 5(e) clearly showed that CTGF levels were dramatically elevated in plasma samples from RILI rats after month 3. However, there was little antagonistic effect on CTGF offered by CTS administration during the entire radiation period. This result was also confirmed by lung tissue immunostaining of CTGF (Figures 5(c) and 5(d)).

### 3.6. CTS Inhibited NOX-4 Activation in RILI Rats

We then evaluate the expressions of NOX-4, NOX-2, STAT3, and COX-2, which were reported to link to TGF-*β*1 production and fibrogenic responses, in the lung tissues. NOX-4 is an important profibrotic signal implicated in aberrant fibroblast activation in idiopathic pulmonary fibrosis [32], and TGF-*β*1-mediated collagen type I gene, *α*-SMA, and fibronectin-1 gene expressions were Nox4-dependent [33, 34]. Additionally, COX-2 is regarded as an antifibrotic enzyme involved in repressing fibroblast-to-myofibroblast differentiation. Reduced COX-2 expression has been observed in patients with idiopathic pulmonary fibrosis [35], and TGF-*β* stimulation lowered levels of COX-2 and thereby contributing to the activation of lung fibroblasts and excessive collagen deposition in pulmonary fibrosis [36].

**Figure 6:**
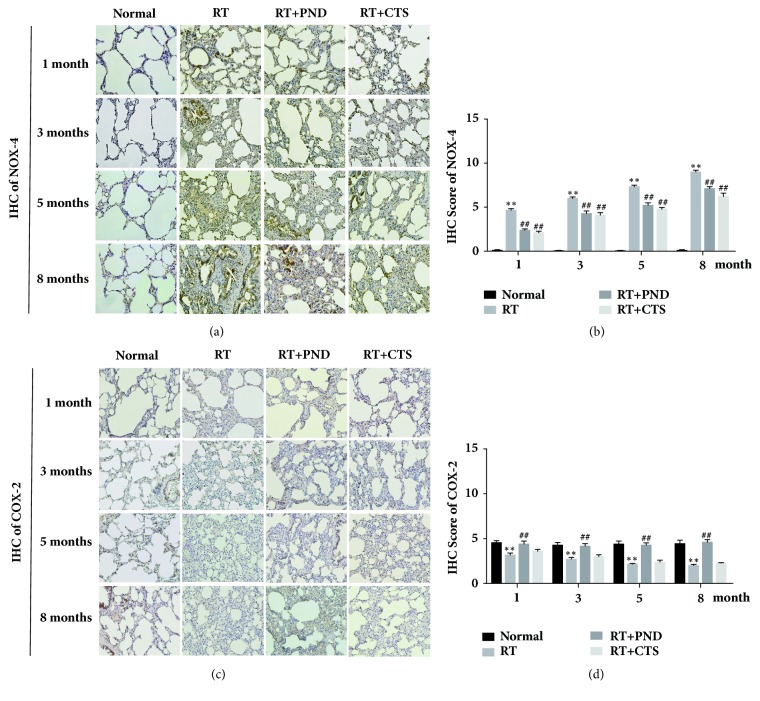
Effects of CTS on NOX-4 and COX-2 levels in RILI rats. (a) IHC staining of NOX-4 in lung tissue sections from each group (magnification × 200). (b) Semiquantitative analysis of NOX-4 IHC staining. (c) IHC staining of COX-2 in lung tissue sections from each group (magnification × 200). (d) Semiquantitative analysis of COX-2 IHC staining.* n* = 6. ^*∗*^*P*< 0.05 and ^*∗∗*^*P* < 0.01 versus Normal; ^#^*P* < 0.05 and ^##^*P* < 0.01 versus RT.

In the present study, we demonstrated a striking difference in the range and distribution of NOX-4 and COX-2 between normal and radiated lung tissues. As shown in Figures 6(a) and 6(b), NOX-4 protein levels were mounting sharply after radiation, both PND and CTS treatments obviously attenuated NOX-4 activation in radiated lung tissues. Also, we noted that CTS suppression was slightly stronger as compared to PND treatment. CTS, however, exhibited little effect on the decreased COX-2 expressions elicited by radiation (Figures 6(c) and 6(d)). Although NOX-2 and STAT3 were partly shown to be implicated in tissue fibrosis, barely the radiated lung tissues present evidence of elevated or reduced NOX-2 and STAT3 expressions (data not shown). Together, CTS could notably attenuate radiation-induced activation of NOX-4 in lung tissues.

### 3.7. CTS Significantly Enhanced MMP-1 Levels in RILI Rats

Fibrosis is pathologically characterized by the excessive matrix accumulation, frequently caused not only by enhanced matrix synthesis, but also by decreased matrix degradation [37]. Matrix metalloproteinases (MMPs) are zinc-dependent endopeptidases that degrade extracellular matrix proteins and thus participate in extracellular matrix remodeling, acting as a powerful brake on the decreased matrix degradation. MMPs superfamily includes multiple types, of which MMP-1 [38, 39], MMP-2 [40], MMP-3 [41], MMP-7 [42], and MMP-9 [40] have been shown to be implicated in the fibrotic process of lung tissues.

**Figure 7:**
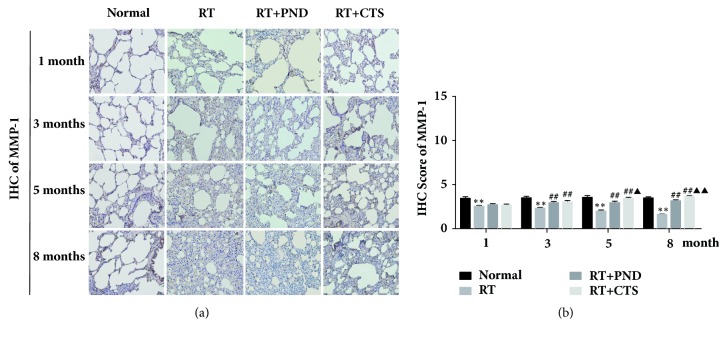
Effects of CTS on MMP-1 levels in RILI rats. (a) IHC staining of MMP-1 in lung tissue sections from each group (magnification × 200). (b) Semiquantitative analysis of MMP-1 IHC staining.* n* = 6. ^*∗*^*P*< 0.05 and ^*∗∗*^*P* < 0.01 versus Normal; ^#^*P* < 0.05 and ^##^*P* < 0.01 versus RT; ^▲^*P*< 0.05 and ^▲▲^*P*< 0.01 versus RT+PND.

To uncover whether CTS affect these enzymes contributing to the degradation of excessive matrix, its effects on MMP-1, MMP-2, MMP-3, MMP-7, and MMP-9 were addressed. In the current experiment, a robust accumulation of collagenous fibers in thickened alveolar septa, particularly after 5-month radiation, was noted in parallel with notable suppressed MMP-1 expressions, as depicted in Figures 7(a) and 7(b). CTS prominently enhanced MMP-1 secretion and, intriguingly, CTS administration significantly outperformed PND on enhancing the enzyme levels. MMP-2, MMP-3, MMP-7, and MMP-9 were reported to be partly involved in tissue fibrosis, while in our work, we found no noticeable change of these enzymes after radiation (data not shown). Together, our findings mirrored CTS's relative superiority over PND in holding the fibrotic process in check, largely, at least in part, by enhancing MMP-1 secretion, which would promote and amplify matrix degradation.

### 3.8. CTS Reduced IL-6 and IL-10 Levels in RILI Rats

**Figure 8:**
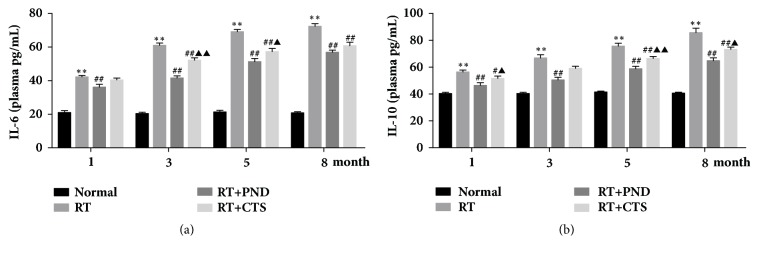
CTS reduced IL-6 and IL-10 levels in RILI rats. (a) Plasma IL-6 level in each group. (b) Plasma IL-10 level in each group.* n* = 6. ^*∗*^*P*< 0.05 and ^*∗∗*^*P* < 0.01 versus Normal; ^#^*P* < 0.05 and ^##^*P* < 0.01 versus RT; ^▲^*P*< 0.05 and ^▲▲^*P*< 0.01 versus RT+PND.

ILs is an important group of inflammatory cytokines, and the production and release of ILs were considered to play critical roles in RILI pathogenesis. To recognize whether inhibition of CTS on radiation-induced inflammatory response was related to critical inflammatory cytokines IL-6 and IL-10, we sought to assess the concentration of the two cytokine in blood plasma. As shown in Figure 8(a), dramatic increases of plasma IL-6 levels were found after 1-month postradiation. Both CTS and PND treatments produced potent inhibition of IL-6 levels.

Radiation also led to significant increase of IL-10 levels, which is inconsistent with some previous studies [43, 44]. We speculate that a multiple role of cytokine IL-10 in inflammation-associated fibrotic process might be responsible for this result [45]. As shown in Figure 8(b), we found a marked drop of IL-10 levels in CTS-treated rats comparing to model RILI rats. Similar to IL-6, PND had a stronger efficacy than CTS in inhibiting cytokine IL-10. While in this study, we found no notably change of IL-17A in both model and treated rats (data not shown). Together, CTS act as an inflammatory inhibitor and its anti-inflammatory effect might be attributed to IL-6 and IL-10 inhibition.

### 3.9. CTS Inhibited CCL3 and CCR1 Activation in RILI Rats

**Figure 9:**
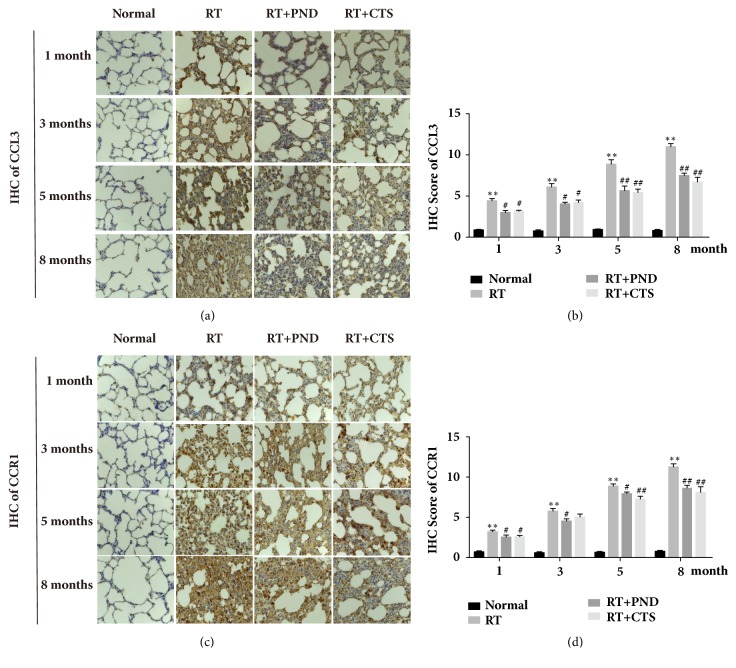
CTS inhibited CCL3 and CCR1 activation in RILI rats. (a) IHC staining of CCL3 in lung tissue sections from each group (magnification × 200). (b) Semiquantitative analysis of CCL3 IHC staining. (c) IHC staining of CCR1 in lung tissue sections from each group (magnification × 200). (d) Semiquantitative analysis of CCR1 IHC staining.* n* = 6. ^*∗*^*P*< 0.05 and ^*∗∗*^*P* < 0.01 versus Normal; ^#^*P* < 0.05 and ^##^*P* < 0.01 versus RT.

Chemokine CCL3 and its receptor CCR1 serve as the key mediators in the dynamic balance of Th1/Th2 in immunocytes, which is crucial in regulating inflammatory and fibrotic responses hooked in RILI process. IHC images depicted that numerous CCL3 and CCR1 positive cells accumulated in the early interstitial inflammation and late fibrosis foci of radiated lung tissues (Figures 9(a) and 9(c)); this finding supports our hypothesis that CCL3/CCR1 activation is implicated in the pathogenic process of RILI.

We also determine whether the accumulation of activated CCL3/CCR1 cells was suppressed in RILI rats after CTS administration. CTS and PND produced marked inhibition of CCL3 and CCR1 levels in lung tissues of RILI rats, ascertained by immunohistochemistry (Figures 9(a)–9(d)). While CTS led to a clear drop of CCR1 levels at month 3 (Figure 9(d)), there was no statistical significance. We speculated that limitation of rat sample size might factor in the nonsignificance. Moreover, in the majority of CTS-treated lung tissues, CCL3/CCR1 inhibition was frequently paralleled with the restoration of pathological abnormalities including inflammatory infiltration and collagen sedimentation. Collectively, CTS could ameliorate radiation-induced interstitial inflammation and fibrosis by its mitigating effect on CCL3/CCR1 activation.

## 4. Discussion 

RILI is mainly characterized by chronic inflammation and the compensatory fibrosis in lung tissue. Since some clinical adverse effects have been observed with long term therapy with PND in RILI patients, the pursuit to discover alternative therapies to treat RILI is of high concern. CTS exhibit a board spectrum of anti-inflammatory properties [24, 25] and show important therapeutic activities in cardiac fibrosis [26]. Therefore, CTS might be a promising agent that selected to reduce radiation-induced lung injury.

In early stage, radiation causes inflammatory response and then stimulates burst of inflammatory cytokines, accompanying with macrophages or neutrophils infiltration in lung tissue. In this study, we demonstrated the infiltration of macrophages and neutrophils and interstitial edema, as well as thickening of alveolar septa in radiated lung tissue. Both CTS and PND treatments could reduce the pathological alterations of chronic inflammation in lung tissue, especially the macrophages and neutrophils infiltration. Corroborating with this, CTS and PND treatments also efficiently restored pulmonary coefficient. Since ILs have a huge role in the pathogenesis of lung inflammatory, we next examined the levels of inflammatory cytokines IL-6 and IL-10. IL-6 was a classic proinflammatory cytokine and its plasma levels were found to increase after radiation in our work. IL-10 is often considered as an anti-inflammatory cytokine, and a couple of works have shown that levels of inflammatory cytokine IL-10 were decreased in rats with radiation-induced lung injury [43, 44]. However, another investigator revealed that the total amount of IL-10 in lung tissue is increased after radiation [45], and lung fibroblasts are able to stimulate the synthesis and release of IL-10 in peripheral blood monocytes [46]; similar results have been observed in our work. IL-10 may play multiple roles in inflammation-associated fibrotic process, since it is an anti-inflammatory cytokine but also a TH2 cytokine with inherent profibrotic effect [45]. Importantly, we found that CTS could inhibit the excessive production and release of IL-6 and IL-10 in RILI rats. PND, by comparison, had a better effect than CTS in ameliorating pulmonary chronic inflammation, as evidenced by pathological examination, pulmonary coefficient, and inflammatory cytokines detection.

In late stage of RILI, pulmonary fibrosis replaced inflammation as the prime concern. We noted that model RILI rats experienced marked fibrotic changes, especially after 5-month radiation. Both CTS and PND alleviated the late stage pulmonary fibrosis to varying degrees. Amazingly, CTS exhibited stronger antifibrosis activity than PND treatment, this result was supported by Masson's trichrome stain as well as by reduced expressions of HYP (fibrosis marker) and *α*-SMA (marker of myofibroblast). Thus, comparing to the PND treatment, the advantage of CTS treatment might be its antifibrosis ability rather than its anti-inflammation activity.

We were further interested to probe the protective mechanisms of CTS against radiation-induced pulmonary fibrosis. Fibrogenic process was due, in part, to enhanced matrix synthesis, proposed to be evoked by multiple profibrotic factors, and, in part, to the decreased matrix degradation. We first measured whether CTS influenced the profibrotic factors CTGF, TGF-*β*1, STAT3, NOX-4, and NOX-2. TGF-*β*1 is long considered to as a central mediator facilitating fibrotic process. TGF-*β*1 stimulation could increase the expression of *α*-SMA [47], and thus accelerate HYP deposition. In addition, NOX-4-dependent generation of hydrogen peroxide (H(2)O(2)) is required for TGF-*β*1-induced myofibroblast differentiation and extracellular matrix production [34]. CTGF serves as another profibrotic signal in tissue remodeling and fibrosis [48], and CTGF blocking was reported to attenuate radiation-induced pulmonary fibrosis [49]. In our work, radiation led to increased levels of the above key profibrotic factors, except for NOX-2 and STAT3, in rats. While effective in reducing the levels of TGF-*β*1 and NOX-4, CTS exhibited little effect on CTGF levels. One possible explanation is that CTGF might not be the therapeutic target of CTS in RILI rats. These findings suggest that CTS could markedly suppress the NOX-4 and TGF-*β*-induced myofibroblast differentiation, thereby reducing the synthesis of collagen, but act independently of CTGF. Although we noted a slight stronger inhibition effect of CTS treatment when compared to PND in this process, no significant differences were achieved.

We then evaluate whether CTS's antifibrotic bioactivity was partially acquired by regulating antifibrotic molecules COX-2, MMP-1, MMP-2, MMP-3, MMP-7, and MMP-9. MMPs are widely acknowledged to act as a powerful brake on excessive matrix deposition through its essential roles in breaking down components of the extracellular matrix. MMPs thus in particular serve as key antifibrotic molecules in protecting against lung fibrosis. COX-2 is also considered as an antifibrotic enzyme involved in repressing fibroblast-to-myofibroblast differentiation, and there was a feedback relationship between matrix stiffening, COX-2 suppression, and fibroblast activation that promotes and amplifies progressive fibrosis [39]. In this experiment, while barely abrogating the COX-2 suppression elicited by radiation-driven matrix stiffening, CTS could prominently enhance the secretion levels of MMP-1. Consistent with trichrome staining analysis, CTS treatment, by contrast, was evidently superior to PND in enhancing MMP-1 levels. These findings raise the intriguing possibility that CTS has a relative advantage over PND in driving the degradation of excessive matrix evoked by radiation exposure and that MMP-1 might be the potential target responsible for CTS's antifibrotic effectiveness.

CCL3 binding to its receptor CCR1 was traditionally emphasized as the central mediators in orchestrating the influx of inflammatory cells into sites of tissue injury [50, 51]. It has recently been reported that a marked increase of CCL3 mRNA expression was observed in inflamed paws, with CCL3 protein detected in neutrophils and macrophages, supporting its involvement in inflammatory responses [52]. The selective interaction of chemokine CCL3 with its receptor, CCR1, is also crucial for thoracic radiation-induced pulmonary fibrosis. Radiated mice develop a marked fibrotic pathology including increased septal thickness and increased collagen deposition, with disturbed lung architecture. By contrast, radiated mice lacking CCL3 or CCR1 had no significant septal thickening and small foci of collagen deposition [53], and CCL3/CCR1 inhibitor could largely prevent fibrogenic process. A similar pivotal role of CCR1 was found in another bleomycin-induced lung fibrosis mouse model [54]. Besides, a targeted null mutation of CCR1 also decreased IL-13-derived inflammation and alveolar remodeling [55]. Another study reported a significant increase in mast cell secretion of key growth factors and cytokines, such as IL-6, TNF-*α*, and TGF*β*-1, upon CCR1-FcepsilonRI costimulation [7]. These observations above indicated that CCL3/CCR1 axle is crucial for promoting secretion of various inflammatory and fibrotic cytokines. In our work, IHC images depicted that numerous CCL3 and CCR1 positive cells accumulated in the early interstitial inflammatory site and late fibrosis foci in radiated lung tissues. Aberrant activation of CCL3/CCR1 orchestrated the influx of inflammatory cells to the injured sites and triggered tissue repair mechanisms, thereby exacerbating the insult and driving pulmonary fibrosis. In contrast, RILI rats treated with CTS developed less CCL3 and CCR1 positive cells in lung tissues. Collectively, the anti-RILI effect of CTS was possibly achieved by regulating the inflammatory and fibrotic cytokines via inhibiting CCL3/CCR1 activation.

Overall, our study on CTS in treating rats with radiation-induced lung injury was encouraging. However, there were also some limitations. For instance, we used only a single dose of CTS, and dose dependence manner of CTS treatment remains unexplored. Besides, the superiority of CTS in antifibrosis ability and its detailed mechanisms merit further investigation in larger sample size studies.

## 5. Conclusion

In summary, CTS could preserve pulmonary function and attenuate radiation-induced lung injury, especially pulmonary fibrosis, in rats. This mitigating effect was partly achieved by suppressing release of inflammatory cytokines IL-6 and IL-10 as well as by balancing levels of fibrotic factors TGF-*β*1, NOX-4, and MMP-1 and by inhibiting CCL3/CCR1 activation. (1) CTS act as an inflammatory inhibitor and its anti-inflammatory effect might be attributed to IL-6 and IL-10 inhibition. (2) In parallel with the reduction of fibrosis, CTS treatment not only effectively diminishes radiation-induced overproduction of profibrotic factors TGF-*β*1 and NOX-4 but also notably rescues the suppression of antifibrotic proteases MMP-1 secretion.

## Data Availability

The data used to support the findings of this study are available from the corresponding author upon request.

## Supplementary Materials

Supplementary Materials (1) Page 1: Weight. Raw data for Figure 1. (2) Page 2-4: Pulmonary Function. Raw data for Figure 2. (3) Page 5-7: H&E and Masson Score, and pulmonary coefficients. Raw data for Figure 3. (4) Page 8-12: *α*-SMA and HYP. Raw data for Figure 4. (5) Page 13-18: TGF-*β*1 and CTGF. Raw data for Figure 5. (6) Page 19-20: NOX-4 and COX-2. Raw data for Figure 6. (7) Page 21: MMP-1. Raw data for Figure 7. (8) Page 22-29: IL-6 and IL-10. Raw data for Figure 8. (9) Page 30-31: CCL3 and CCR1. Raw data for Figure 9.
