# Supplementary material for: Cryptotanshinone Ameliorates Radiation-Induced Lung Injury in Rats
Source: Evid Based Complement Alternat Med. 2019 Feb 20;2019:1908416. doi: 10.1155/2019/1908416 (PMC6402207; doi:10.1155/2019/1908416)
Supplement: Supplementary Materials — (1) Page 1: Weight. Raw data for Figure 1. (2) Page 2-4: Pulmonary Function. Raw data for Figure 2. (3) Page 5-7: H&E and Masson Score, and pulmonary coefficients. Raw data for Figure 3. (4) Page 8-12: α-SMA and HYP. Raw data for Figure 4. (5) Page 13-18: TGF-β1 and CTGF. Raw data for Figure 5. (6) Page 19-20: NOX-4 and COX-2. Raw data for Figure 6. (7) Page 21: MMP-1. Raw data for Figure 7. (8) Page 22-29: IL-6 and IL-10. Raw data for Figure 8. (9) Page 30-31: CCL3 and CCR1. Raw data for Figure 9. [file 1908416.f1.pdf]

A description for supplementary files

1) Page 1: Weight. Raw data for Fig. 1.

2) Page 2-4: Pulmonary Function. Raw data for Fig. 2.

3) Page 5-7: H&E and Masson Score, and Pulmonary coefficients. Raw data for Fig. 3.

4) Page 8-12: α-SMA and HYP. Raw data for Fig. 4.

5) Page 13-18: TGF-β1 and CTGF. Raw data for Fig. 5.

6) Page 19-20: NOX-4 and COX-2. Raw data for Fig. 6.

7) Page 21: MMP-1. Raw data for Fig. 7.

8) Page 22-29: IL-6 and IL-10. Raw data for Fig. 8.

9) Page 30-31: CCL3 and CCR1. Raw data for Fig. 9.

Body Weight（g）										
Group	Sign	Time（month）								
		Before medication	1	2	3	4	5	6	7	8
Normal	left ear	198	222	298	346	379	418	458	492	523
	right ear	201	253	326	355	392	449	457	472	494
	left eye	205	252	319	363	411	462	486	516	533
	right eye	219	268	314	337	378	421	452	483	517
	left front paw	196	246	295	334	384	439	458	464	471
	right front paw	202	299	347	389	414	459	472	480	496
RT	left ear	200	235	247	258	269	286	309	316	334
	right ear	207	265	295	308	324	347	362	399	410
	left eye	216	278	343	357	365	382	409	422	439
	right eye	188	202	225	239	252	265	289	302	327
	left front paw	220	225	279	282	295	317	330	352	385
	right front paw	213	245	289	315	332	348	357	381	409
RT+PND	left ear	210	261	323	374	398	418	456	487	499
	right ear	193	246	293	342	370	406	436	469	503
	left eye	185	204	220	260	325	345	372	406	421
	right eye	216	250	327	362	409	456	478	495	517
	left front paw	210	260	308	335	362	398	415	446	473
	right front paw	215	308	356	383	403	426	459	482	507
RT+CTS	left ear	210	236	261	290	352	383	416	438	462
	right ear	193	253	317	353	406	426	469	483	501
	left eye	206	217	258	279	323	353	381	411	445
	right eye	199	242	320	351	382	421	436	462	491
	left front paw	214	284	345	392	420	449	478	492	517
	right front paw	200	232	275	324	376	423	451	479	506

Respiratory Rate							
Group	Sign	Time（month）					
		1	2	3	4	5	8
Normal	left ear	102	119	107	102	118	105
	right ear	90	112	119	116	109	111
	left eye	95	109	115	97	106	119
	right eye	98	111	109	122	124	107
	left front paw	112	106	121	119	114	103
	right front paw	100	93	97	96	98	102
RT	left ear	117	122	134	140	155	169
	right ear	105	113	138	159	161	175
	left eye	108	111	135	147	152	166
	right eye	109	118	141	167	183	195
	left front paw	91	106	148	162	176	189
	right front paw	106	115	146	154	164	177
RT+PND	left ear	102	108	123	127	126	125
	right ear	98	116	126	129	127	119
	left eye	107	109	131	135	124	121
	right eye	92	102	135	130	125	122
	left front paw	102	115	139	140	138	136
	right front paw	97	100	126	125	118	123
RT+CTS	left ear	109	113	132	129	118	124
	right ear	106	105	127	124	123	119
	left eye	98	115	139	131	127	120
	right eye	105	114	136	128	120	124
	left front paw	98	108	130	123	117	106
	right front paw	90	101	124	129	121	113

Tidal Volume							
Group	Sign	Time（month）					
		1	2	3	4	5	8
Normal	left ear	0.86	0.83	0.82	1.04	0.81	0.95
	right ear	0.78	0.88	0.85	0.98	1.05	0.98
	left eye	0.83	0.86	0.89	0.82	0.93	1.12
	right eye	1.01	0.99	1.03	0.93	0.86	0.79
	left front paw	1.05	0.98	0.92	0.85	0.84	0.95
	right front paw	0.92	0.86	0.95	0.89	1.04	0.85
RT	left ear	0.83	0.8	0.79	0.56	0.58	0.59
	right ear	0.75	0.73	0.72	0.44	0.43	0.49
	left eye	0.82	0.8	0.67	0.39	0.47	0.48
	right eye	0.98	0.85	0.65	0.46	0.52	0.5
	left front paw	0.81	0.78	0.72	0.52	0.49	0.36
	right front paw	0.78	0.73	0.73	0.55	0.42	0.45
RT+PND	left ear	0.93	0.85	0.76	0.74	0.68	0.73
	right ear	0.86	0.91	0.75	0.75	0.78	0.79
	left eye	0.75	0.72	0.79	0.73	0.62	0.69
	right eye	0.97	0.84	0.81	0.84	0.83	0.81
	left front paw	0.87	0.83	0.86	0.95	0.91	0.84
	right front paw	0.92	0.89	0.83	0.85	0.89	0.82
RT+CTS	left ear	0.98	0.93	0.82	0.78	0.84	0.85
	right ear	0.79	0.82	0.8	0.79	0.78	0.82
	left eye	0.84	0.8	0.76	0.85	0.75	0.79
	right eye	0.85	0.86	0.83	0.78	0.75	0.84
	left front paw	0.9	0.83	0.82	0.83	0.82	0.91
	right front paw	0.82	0.86	0.73	0.77	0.88	0.93

Maximum Voluntary Ventilation							
Group	Sign	Time（month）					
		1	2	3	4	5	8
Normal	left ear	133.82	125.88	135.26	145.53	137.99	128.75
	right ear	127.55	123	122.83	130.55	135.27	137.55
	left eye	140.89	133.41	129.34	137.62	146.27	149.72
	right eye	138.12	132.48	140.67	142.56	125.83	138.25
	left front paw	120.39	123.76	148.65	139.07	128.19	124.82
	right front paw	148.63	139.53	137.41	142.35	149.66	141.42
RT	left ear	123.85	115.76	101.34	88.43	82.46	80.29
	right ear	136.32	127.65	97.22	84.88	80.45	79.43
	left eye	127.32	118.27	96.98	85.27	83.22	78.67
	right eye	118.15	105.68	94.69	86.49	84.34	80.51
	left front paw	135.21	128.45	101.34	93.44	90.57	83.34
	right front paw	129.67	122.55	106.38	84.21	80.66	75.35
RT+PND	left ear	123.98	126.23	114.43	108.55	115.23	109.33
	right ear	129.54	123.78	109.24	120.01	121.23	115.86
	left eye	139.32	133.48	111.26	125.34	105.31	101.66
	right eye	132.58	129.45	112.67	113.49	116.88	112.51
	left front paw	135.59	129.33	106.93	99.94	112.43	108.25
	right front paw	127.51	115.77	103.35	110.22	102.69	114.76
RT+CTS	left ear	136.44	125.79	108.57	115.61	120.86	117.54
	right ear	125.12	122.55	105.82	123.49	125.15	119.95
	left eye	121.68	120.25	112.34	109.67	116.34	123.26
	right eye	128.21	117.37	91.35	114	113.54	125.73
	left front paw	137.59	134.72	118.11	117.48	110.66	118.41
	right front paw	129.53	127.36	116.42	112.3	107.39	113.21

H&E																	
Group	Sign	Time（month）															
		1				3				5				8			
		No.1 visual	No.2 visual	No.3 visual	Mean	No.1 visual	No.2 visual	No.3 visual	Mean	No.1 visual	No.2 visual	No.3 visual	Mean	No.1 visual	No.2 visual	No.3 visual	Mean
Normal	left ear	0	0	1	0.33	1	0	0	0.33	0	0	0	0	0	1	0	0.33
	right ear	0	0	0	0	0	0	0	0	0	0	0	0	0	0	0	0
	left eye	0	0	0	0	1	0	0	0.33	0	0	0	0	0	0	0	0
	right eye	1	0	0	0.33	0	0	0	0	0	0	0	0	0	0	0	0
	left front paw	0	0	0	0	0	1	0	0.33	0	0	0	0	0	0	0	0
	right front paw	0	0	0	0	0	0	0	0	0	0	1	0.33	0	0	0	0
RT	left ear	1	1	1	1	2	2	2	2	3	3	2	2.67	3	3	3	3
	right ear	1	1	1	1	2	2	2	2	2	2	3	2.33	3	2	3	2.67
	left eye	1	1	1	1	2	1	2	1.67	3	3	2	2.67	3	3	3	3
	right eye	2	1	1	1.33	2	2	3	2.33	3	2	3	2.67	3	3	3	3
	left front paw	1	1	1	1	3	2	2	2.33	2	3	3	2.67	3	3	3	3
	right front paw	1	2	1	1.33	2	2	1	1.67	3	3	2	2.67	3	3	3	3
RT+PND	left ear	0	0	1	0.33	2	1	2	1.67	2	2	2	2	2	3	2	2.33
	right ear	1	0	0	0.33	1	2	2	1.67	2	2	3	2.33	3	2	2	2.33
	left eye	0	0	1	0.33	1	1	1	1	2	1	2	1.67	2	2	1	1.67
	right eye	0	1	0	0.33	2	2	2	2	2	1	2	1.67	2	2	2	2
	left front paw	1	0	1	0.67	2	1	1	1.33	1	2	2	1.67	2	2	2	2
	right front paw	1	0	2	1	1	2	2	1.67	2	2	3	2.33	2	2	2	2
RT+CTS	left ear	1	1	0	0.67	2	2	2	2	2	3	2	2.33	2	1	2	1.67
	right ear	0	1	1	0.67	1	1	2	1.33	1	2	2	1.67	2	2	1	1.67
	left eye	1	0	1	0.67	1	2	1	1.33	2	2	1	1.67	2	2	2	2
	right eye	1	0	1	0.67	2	2	2	2	2	1	2	1.67	3	2	2	2.33
	left front paw	0	1	0	0.33	2	2	1	1.67	2	2	3	2.33	2	1	2	1.67
	right front paw	1	0	1	0.67	1	2	1	1.33	1	2	2	1.67	2	2	2	2

Masson																	
Group	Sign	Time（month）															
		1				3				5				8			
		No.1 visual	No.2 visual	No.3 visual	Mean	No.1 visual	No.2 visual	No.3 visual	Mean	No.1 visual	No.2 visual	No.3 visual	Mean	No.1 visual	No.2 visual	No.3 visual	Mean
Normal	left ear	0	0	0	0	0	0	0	0	0	0	0	0	0	0	0	0
	right ear	0	0	1	0.33	1	0	0	0.33	0	0	0	0	0	0	0	0
	left eye	0	0	0	0	0	0	0	0	1	0	0	0.33	0	0	0	0
	right eye	0	0	0	0	1	0	1	0.67	0	0	0	0	0	0	0	0
	left front paw	0	0	0	0	0	0	0	0	1	0	1	0.67	0	1	0	0.33
	right front paw	0	0	0	0	0	0	0	0	0	1	0	0.33	0	0	0	0
RT	left ear	1	0	0	0.33	0	1	1	0.67	2	2	3	2.33	3	3	3	3
	right ear	0	1	0	0.33	0	0	1	0.33	1	2	1	1.33	3	3	3	3
	left eye	1	0	0	0.33	1	1	1	1	3	2	2	2.33	2	3	3	2.67
	right eye	0	0	1	0.33	0	1	1	0.67	2	2	1	1.67	3	3	3	3
	left front paw	0	1	1	0.67	1	1	1	1	2	2	2	2	3	3	3	3
	right front paw	1	0	0	0.33	0	1	0	0.33	3	2	2	2.33	3	3	3	3
RT+PND	left ear	1	0	0	0.33	0	0	1	0.33	2	2	1	1.67	3	3	2	2.67
	right ear	0	0	0	0	0	0	1	0.33	2	1	2	1.67	2	2	2	2
	left eye	0	1	0	0.33	0	0	0	0	1	1	2	1.33	2	3	2	2.33
	right eye	1	0	0	0.33	0	0	0	0	1	2	2	1.67	2	2	2	2
	left front paw	1	0	0	0.33	1	1	0	0.67	1	2	1	1.33	3	2	3	2.67
	right front paw	0	1	0	0.33	0	0	1	0.33	1	2	1	1.33	3	3	3	3
RT+CTS	left ear	0	1	1	0.67	1	0	0	0.33	2	1	1	1.33	2	2	2	2
	right ear	0	1	1	0.67	1	1	0	0.67	1	0	1	0.67	1	1	2	1.33
	left eye	1	0	0	0.33	0	0	1	0.33	2	1	1	1.33	2	2	2	2
	right eye	0	0	0	0	0	0	0	0	2	1	1	1.33	1	2	2	1.67
	left front paw	1	0	0	0.33	1	0	0	0.33	1	0	1	0.67	2	2	2	2
	right front paw	0	0	0	0	0	0	0	0	1	2	1	1.33	2	2	1	1.67

Pulmonary Coefficient							
Group	Sign	Time（month）					
		1	2	3	4	5	8
Normal	left ear	6.58	6.89	6.75	5.65	6.13	5.14
	right ear	6.46	6.34	6.37	6.96	5.18	6.69
	left eye	6.97	6.17	6.65	5.68	6.81	5.45
	right eye	6.98	5.77	6.42	6.34	5.54	5.83
	left front paw	6.61	5.99	5.23	6.39	6.53	5.48
	right front paw	5.83	6.69	5.98	6.15	6.65	5.29
RT	left ear	10.62	12.71	12.83	13.15	13.35	13.95
	right ear	10.76	10.87	11.63	11.84	12.75	13.63
	left eye	9.54	9.81	9.95	10.65	12.02	14.11
	right eye	11.13	11.79	12.32	12.89	13.34	13.75
	left front paw	11.69	12.55	12.97	13.26	13.78	14.28
	right front paw	10.45	11.88	11.95	12.44	13.42	14.65
RT+PND	left ear	9.59	9.64	8.79	8.51	8.09	7.62
	right ear	8.42	8.58	8.26	8.02	7.88	7.01
	left eye	10.57	9.88	9.67	8.92	8.63	7.86
	right eye	9.16	8.92	8.69	8.29	7.92	7.48
	left front paw	9.85	9.63	8.47	7.88	7.65	6.93
	right front paw	10.61	9.57	8.98	8.71	8.52	7.97
RT+CTS	left ear	9.77	9.64	8.92	8.37	7.84	6.98
	right ear	9.98	9.42	9.06	8.83	8.15	6.86
	left eye	9.49	9.27	8.81	8.07	7.56	6.76
	right eye	9.54	9.17	8.72	7.84	7.21	6.64
	left front paw	10.52	9.87	9.74	8.93	8.46	7.67
	right front paw	10.69	9.82	9.68	9.23	8.89	6.42

α-SMA																	
Time（month）																	
		1				3				5				8			
Group	Sign	No.1 visual	No.2 visual	No.3 visual	Mean	No.1 visual	No.2 visual	No.3 visual	Mean	No.1 visual	No.2 visual	No.3 visual	Mean	No.1 visual	No.2 visual	No.3 visual	Mean
Normal	left ear	1	0	1	0.67	1	1	1	1	0	1	1	0.67	1	0	1	0.67
	right ear	1	1	1	1	0	0	1	0.33	0	1	0	0.33	1	1	1	1
	left eye	0	1	0	0.33	0	1	1	0.67	1	1	1	1	1	0	1	0.67
	right eye	0	1	1	0.67	1	0	1	0.67	1	0	0	0.33	0	1	1	0.67
	left front paw	1	1	0	0.67	1	1	1	1	0	1	1	0.67	1	1	0	0.67
	right front paw	1	0	1	0.67	1	1	0	0.67	1	1	0	0.67	1	1	1	1
RT	left ear	3	2	2	2.33	3	2	2	2.33	4	4	4	4	3	4	4	3.67
	right ear	2	2	1	1.67	3	3	2	2.67	4	3	3	3.33	4	4	4	4
	left eye	2	1	2	1.67	3	4	4	3.67	3	3	4	3.33	4	4	3	3.67
	right eye	2	3	2	2.33	4	3	3	3.33	4	4	6	4.67	4	6	4	4.67
	left front paw	2	2	2	2	2	3	2	2.33	3	4	4	3.67	3	4	4	3.67
	right front paw	2	3	2	2.33	3	3	3	3	3	3	3	3	4	4	6	4.67
RT+PND	left ear	1	1	2	1.33	2	1	2	1.67	2	3	3	2.67	3	2	3	2.67
	right ear	2	2	1	2	1	2	1	1.33	3	2	2	2.33	2	2	3	2.33
	left eye	2	1	2	1.67	2	1	1	1.33	1	2	1	1.33	3	3	3	3
	right eye	1	2	2	1.67	1	2	2	1.67	3	3	2	2.67	3	3	4	3.33
	left front paw	2	2	2	2	2	2	2	2	2	2	1	1.67	3	3	3	3
	right front paw	2	1	1	1.33	2	1	2	1.67	3	2	2	2.33	3	3	2	2.67
RT+CTS	left ear	2	2	1	1.67	2	1	1	1.33	2	1	2	1.67	2	2	2	2
	right ear	1	2	2	1.67	2	2	2	2	2	2	1	1.67	2	3	2	2.33
	left eye	1	1	1	1	1	2	2	1.67	2	1	2	1.67	2	2	2	2
	right eye	2	1	2	1.67	1	1	1	1	2	2	2	2	3	2	2	2.33
	left front paw	1	2	2	1.67	1	1	2	1.33	2	2	2	2	2	2	2	2
	right front paw	2	2	2	2	1	2	1	1.33	3	2	2	2.33	3	2	2	2.33

HYP-1month

		OD Values						Concentration						Concentration(average)	SEM	epeat of biolog	P value vs. RT	P value vs. RT+PND
Name	Group	1	2	3	4	5	6	1	2	3	4	5	6					
HYP	Normal	0.08901	0.08906	0.08901	0.08892	0.08911	0.08898	0.389	0.406	0.389	0.354	0.424	0.375	0.39	0.01	6	<0.01	
	RT	0.08918	0.08943	0.08914	0.08910	0.08931	0.08949	0.452	0.543	0.437	0.422	0.498	0.566	0.49	0.02	6		
	RT+PND	0.08900	0.08909	0.08918	0.08915	0.08885	0.08924	0.385	0.418	0.451	0.441	0.329	0.473	0.42	0.02	6	<0.05	
	RT+CTS	0.08902	0.08899	0.08925	0.08919	0.08898	0.08921	0.392	0.381	0.477	0.456	0.375	0.463	0.42	0.02	6	<0.05	ns

HYP-3month

		OD Values						Concentration						Concentration(average)	SEM	epeat of biolog	P value vs. RT	P value vs. RT+PND
Name	Group	1	2	3	4	5	6	1	2	3	4	5	6					
HYP	Normal	0.08918	0.08902	0.08902	0.08897	0.08914	0.08907	0.452	0.391	0.393	0.373	0.436	0.409	0.41	0.01	6	<0.01	
	RT	0.08924	0.08950	0.08920	0.08931	0.08933	0.08951	0.474	0.569	0.458	0.497	0.506	0.573	0.51	0.02	6		
	RT+PND	0.08919	0.08910	0.08916	0.08923	0.08893	0.08926	0.453	0.42	0.445	0.468	0.357	0.482	0.44	0.02	6	<0.01	
	RT+CTS	0.08910	0.08915	0.08930	0.08928	0.08901	0.08930	0.422	0.438	0.494	0.488	0.386	0.496	0.45	0.02	6	<0.05	ns

HYP-5month

		OD Values						Concentration						Concentration(average)	SEM	epeat of biolog	P value vs. RT	P value vs. RT+PND
Name	Group	1	2	3	4	5	6	1	2	3	4	5	6					
HYP	Normal	0.08911	0.08905	0.08901	0.08899	0.08909	0.08907	0.426	0.402	0.386	0.382	0.417	0.411	0.40	0.01	6	<0.01	
	RT	0.08949	0.08955	0.08924	0.08951	0.08941	0.08955	0.565	0.588	0.472	0.572	0.536	0.588	0.55	0.02	6		
	RT+PND	0.08927	0.08922	0.08931	0.08924	0.08901	0.08941	0.483	0.466	0.499	0.474	0.388	0.536	0.47	0.02	6	<0.01	
	RT+CTS	0.08916	0.08923	0.08925	0.08918	0.08905	0.08935	0.443	0.469	0.478	0.449	0.403	0.514	0.46	0.02	6	<0.01	ns

HYP-8month																		
		OD Values						Concentration						Concentration(average)	SEM	epeat of biolog	P value vs. RT	P value vs. RT+PND
Name	Group	1	2	3	4	5	6	1	2	3	4	5	6					
HYP	Normal	0.08909	0.08908	0.08896	0.08896	0.08913	0.08912	0.418	0.413	0.371	0.368	0.434	0.427	0.41	0.01	6	<0.01	
	RT	0.08960	0.08968	0.08941	0.08962	0.08954	0.08965	0.606	0.637	0.534	0.612	0.584	0.624	0.60	0.01	6		
	RT+PND	0.08933	0.08917	0.08938	0.08929	0.08905	0.08938	0.505	0.447	0.523	0.493	0.403	0.525	0.48	0.02	6	<0.01	
	RT+CTS	0.08923	0.08911	0.08932	0.08913	0.08912	0.08938	0.471	0.426	0.503	0.432	0.427	0.526	0.46	0.02	6	<0.01	ns

TGF-β1 IHC																	
Time（month）																	
		1				3				5				8			
Group	Sign	No.1 visual	No.2 visual	No.3 visual	Mean	No.1 visual	No.2 visual	No.3 visual	Mean	No.1 visual	No.2 visual	No.3 visual	Mean	No.1 visual	No.2 visual	No.3 visual	Mean
Normal	left ear	1	1	0	0.67	0	1	0	0.33	1	2	0	1	0	1	1	0.67
	right ear	0	1	0	0.33	1	1	0	0.67	0	1	1	0.67	1	1	0	0.67
	left eye	0	0	0	0	1	0	0	0.33	1	1	0	0.67	0	1	0	0.33
	right eye	1	0	1	0.67	0	1	1	0.67	0	0	1	0.33	1	0	0	0.33
	left front paw	0	1	0	0.33	1	1	1	1	1	0	1	0.67	1	1	0	0.67
	right front paw	0	1	1	0.67	1	1	0	0.67	0	1	0	0.33	0	1	1	0.67
RT	left ear	4	6	6	5.33	6	8	8	7.33	12	12	9	11	12	9	12	11
	right ear	6	8	9	7.67	8	8	12	9.33	9	9	12	10	9	12	12	11
	left eye	6	8	6	6.67	9	9	9	9	8	12	9	9.67	12	12	12	12
	right eye	6	8	9	7.67	6	8	12	8.67	8	9	8	8.33	12	9	12	11
	left front paw	9	6	6	7	8	6	9	7.67	9	8	9	8.67	9	12	9	10
	right front paw	8	6	6	6.67	12	9	9	10	12	9	9	10	12	12	9	11
RT+PND	left ear	4	2	2	2.67	4	6	4	4.67	6	6	8	6.67	8	6	9	7.67
	right ear	2	4	4	3.33	6	2	4	4	8	8	6	7.33	6	9	9	8
	left eye	2	3	2	2.33	4	4	4	4	3	4	6	4.33	6	6	6	6
	right eye	2	2	4	2.67	6	3	6	5	6	3	6	5	9	8	9	8.67
	left front paw	2	3	4	3	4	2	4	3.33	8	4	6	6	9	9	9	9
	right front paw	3	4	2	3	4	6	2	4	6	3	4	4.33	8	9	9	8.67
RT+CTS	left ear	4	2	2	2.67	3	6	4	4.33	4	3	6	4.33	8	8	9	8.33
	right ear	2	3	2	2.33	6	2	4	4	6	4	4	4.67	6	8	6	6.67
	left eye	4	2	4	3.33	4	4	6	4.67	3	4	6	4.33	9	6	9	8
	right eye	3	2	2	2.33	3	6	3	4	6	4	4	4.67	9	8	6	7.67
	left front paw	2	4	4	3.33	4	6	4	4.67	4	6	4	4.67	8	6	6	6.67
	right front paw	4	2	4	3.33	6	4	4	4.67	8	6	6	6.67	6	4	8	6

CTGF																	
**Time（month）**																	
		1				3				5				8			
Group	Sign	No.1 visual	No.2 visual	No.3 visual	Mean	No.1 visual	No.2 visual	No.3 visual	Mean	No.1 visual	No.2 visual	No.3 visual	Mean	No.1 visual	No.2 visual	No.3 visual	Mean
Normal	left ear	0	1	1	0.67	0	0	0	0	0	0	0	0	1	0	1	0.67
	right ear	0	0	1	0.33	0	1	0	0.33	0	0	1	0.33	0	0	0	0
	left eye	0	0	0	0	0	0	0	0	0	1	0	0.33	0	1	0	0.33
	right eye	1	0	1	0.67	0	0	0	0	0	0	0	0	1	0	0	0.33
	left front paw	0	0	0	0	0	1	0	0.33	1	0	0	0.33	0	0	0	0
	right front	0	1	0	0.33	0	0	0	0	0	0	0	0	0	0	0	0
RT	left ear	2	1	1	1.33	4	3	3	3.33	4	4	6	4.67	8	8	9	8.33
	right ear	1	1	1	1	3	3	4	3.33	4	6	4	4.67	6	6	8	6.67
	left eye	1	2	2	1.67	4	4	3	3.67	4	4	6	4.67	8	8	9	8.33
	right eye	2	1	1	1.33	3	3	4	3.33	6	4	6	5.33	6	6	6	6
	left front paw	1	2	1	1.33	3	3	3	3	8	6	6	6.67	9	9	8	8.67
	right front	1	2	1	1.33	3	4	4	3.67	4	6	4	4.67	9	8	8	8.33
RT+PND	left ear	2	1	1	1.33	4	3	3	3.33	4	6	6	5.33	4	6	6	5.33
	right ear	1	1	1	1	3	3	3	3	4	4	3	3.67	6	8	6	6.67
	left eye	1	2	1	1.33	3	4	3	3.33	3	4	4	3.67	6	8	8	7.33
	right eye	1	1	2	1.33	4	3	4	3.67	4	3	4	3.67	4	4	6	4.67
	left front paw	1	1	0	0.67	3	3	3	3	4	4	4	4	6	6	6	6
	right front	1	1	1	1	3	4	3	3.33	3	4	3	3.33	8	6	4	6
RT+CTS	left ear	1	1	1	1	3	3	3	3	4	4	6	4.67	8	8	6	7.33
	right ear	1	2	1	1.33	4	3	3	3.33	6	6	4	5.33	6	8	6	6.67
	left eye	1	2	1	1.33	3	4	4	3.67	4	4	4	4	6	6	8	6.67
	right eye	2	1	1	1.33	3	3	4	3.33	4	6	6	5.33	8	8	6	7.33
	left front paw	1	1	2	1.33	4	4	3	3.67	4	4	6	4.67	8	6	6	6.67
	right front	1	1	1	1	3	3	3	3	6	4	4	4.67	6	6	8	6.67

CTGF-1month																		
		OD Values						Concentration						Concentration(average)	SEM	epeat of biolog	P value vs. RT	P value vs. RT+PND
Name	Group	1	2	3	4	5	6	1	2	3	4	5	6					
CTGF	Normal	0.50022	0.50774	0.53640	0.50596	0.48930	0.47849	152.63	155.42	166.05	154.76	148.58	144.57	153.67	2.99	6	ns	
	RT	0.52106	0.55500	0.52939	0.51661	0.49777	0.51453	160.36	172.95	163.45	158.71	151.72	157.94	160.86	2.89	6		
	RT+PND	0.47609	0.52766	0.50639	0.50373	0.48184	0.51179	143.68	162.81	154.92	153.93	145.81	156.92	153.01	2.91	6	ns	
	RT+CTS	0.49521	0.50470	0.53181	0.50785	0.49761	0.51863	150.77	154.29	164.35	155.46	151.66	159.46	156.00	2.09	6	ns	ns

CTGF-3month

		OD Values						Concentration						Concentration(average)	SEM	repeat of biolog	P value vs. RT	P value vs. RT+PND
Name	Group	1	2	3	4	5	6	1	2	3	4	5	6					
CTGF	Normal	0.50397	0.46364	0.49809	0.47569	0.58821	0.57969	154.02	139.06	151.84	143.53	185.27	182.11	159.31	8.03	6	<0.01	
	RT	0.95794	0.98374	1.11194	1.07094	0.89395	0.85020	322.43	332	379.56	364.35	298.69	282.46	329.92	15.21	6		
	RT+PND	0.91796	0.86551	0.83761	0.91901	0.85723	0.88022	307.6	288.14	277.79	307.99	285.07	293.6	293.37	5.02	6	ns	
	RT+CTS	0.94818	0.93282	0.90254	0.84588	0.91608	0.86378	318.81	313.11	301.88	280.86	306.9	287.5	301.51	6.02	6	ns	ns

CTGF-5month

		OD Values						Concentration						Concentration(average)	SEM	epeat of biolog	P value vs. RT	P value vs. RT+PND
Name	Group	1	2	3	4	5	6	1	2	3	4	5	6					
CTGF	Normal	0.51014	0.46086	0.47817	0.52232	0.49041	0.58988	156.31	138.03	144.45	160.83	148.99	185.89	155.75	6.88	6	<0.01	
	RT	1.01053	1.04490	1.07647	0.99943	0.99374	1.10787	341.94	354.69	366.4	337.82	335.71	378.05	352.44	6.98	6		
	RT+PND	0.96411	0.93845	0.92023	1.00342	0.95047	0.98312	324.72	315.2	308.44	339.3	319.66	331.77	323.18	4.58	6	<0.01	
	RT+CTS	1.04528	1.03129	0.99724	0.96969	1.01913	0.95260	354.83	349.64	337.01	326.79	345.13	320.45	338.98	5.47	6	ns	ns

CTGF-8month																		
		OD Values						Concentration						Concentration(average)	SEM	epeat of biolog	P value vs. RT	P value vs. RT+PND
Name	Group	1	2	3	4	5	6	1	2	3	4	5	6					
CTGF	Normal	0.56812	0.42032	0.49518	0.46105	0.55971	0.52343	177.82	122.99	150.76	138.1	174.7	161.24	154.27	8.70	6	<0.01	
	RT	1.06830	1.11402	1.11989	1.07903	1.06000	1.13647	363.37	380.33	382.51	367.35	360.29	388.66	373.75	4.73	6		
	RT+PND	1.05892	1.05938	0.97880	1.07488	0.98266	1.03843	359.89	360.06	330.17	365.81	331.6	352.29	349.97	6.29	6	<0.05	
	RT+CTS	1.12674	1.07655	1.08466	1.00916	1.11237	1.05129	385.05	366.43	369.44	341.43	379.72	357.06	366.52	6.44	6	ns	ns

NOX-4																	
Time（month）																	
		1				3				5				8			
Group	Sign	No.1 visual	No.2 visual	No.3 visual	Mean	No.1 visual	No.2 visual	No.3 visual	Mean	No.1 visual	No.2 visual	No.3 visual	Mean	No.1 visual	No.2 visual	No.3 visual	Mean
Normal	left ear	0	0	0	0	0	0	0	0	0	0	0	0	1	0	0	0.33
	right ear	1	0	0	0.33	0	0	0	0	0	1	0	0.33	0	0	0	0
	left eye	0	0	1	0.33	0	0	0	0	0	0	0	0	0	0	0	0
	right eye	0	0	0	0	0	0	0	0	0	0	0	0	0	0	0	0
	left front paw	0	0	0	0	0	0	0	0	0	0	0	0	0	0	0	0
	right front paw	0	0	0	0	1	0	0	0.33	0	0	0	0	0	1	0	0.33
RT	left ear	4	6	4	4.67	6	6	6	6	8	8	6	7.33	9	9	9	9
	right ear	4	4	4	4.00	4	6	6	5.33	6	6	8	6.67	9	9	8	8.67
	left eye	6	4	4	4.67	6	6	6	6	8	6	8	7.33	8	9	9	8.67
	right eye	6	6	4	5.33	6	8	6	6.67	8	8	6	7.33	9	8	9	8.67
	left front paw	4	6	4	4.67	6	6	6	6	6	8	8	7.33	9	9	9	9
	right front paw	4	4	6	4.67	6	6	6	6	8	8	8	8	9	12	9	10
RT+PND	left ear	3	3	2	2.67	4	4	6	4.67	6	4	4	4.67	6	8	6	6.67
	right ear	3	3	3	3	4	6	4	4.67	4	4	6	4.67	8	8	6	7.33
	left eye	2	2	2	2	3	3	4	3.33	6	6	4	5.33	8	8	8	8
	right eye	2	3	2	2.33	6	4	6	5.33	6	6	6	6	8	6	6	6.67
	left front paw	2	2	3	2.33	4	4	4	4	4	6	4	4.67	8	8	6	7.33
	right front paw	2	2	2	2	4	4	3	3.67	6	6	6	6	6	8	6	6.67
RT+CTS	left ear	2	2	1	1.67	4	4	4	4	4	4	6	4.67	6	6	6	6
	right ear	2	3	2	2.33	6	4	4	4.67	6	4	6	5.33	8	6	8	7.33
	left eye	2	1	2	1.67	4	4	6	4.67	4	4	4	4	6	4	6	5.33
	right eye	2	2	2	2	4	6	4	4.67	4	6	4	4.67	4	6	6	5.33
	left front paw	3	2	3	2.67	3	4	3	3.33	6	4	4	4.67	6	8	8	7.33
	right front paw	3	2	2	2.33	3	3	4	3.33	4	6	6	5.33	6	6	6	6

COX-2																	
**Time（month）**																	
		1				3				5				8			
Group	Sign	No.1 visual	No.2 visual	No.3 visual	Mean	No.1 visual	No.2 visual	No.3 visual	Mean	No.1 visual	No.2 visual	No.3 visual	Mean	No.1 visual	No.2 visual	No.3 visual	Mean
Normal	left ear	6	4	3	4.33	3	3	4	3.33	4	4	3	3.67	4	6	4	4.67
	right ear	6	6	4	5.33	6	4	6	5.33	6	6	4	5.33	3	3	3	3
	left eye	4	4	3	3.67	4	4	3	3.67	3	4	4	3.67	6	6	4	5.33
	right eye	4	6	4	4.67	6	4	4	4.67	4	4	6	4.67	4	4	3	3.67
	left front paw	6	4	4	4.67	4	6	4	4.67	4	6	6	5.33	6	4	4	4.67
	right front paw	4	4	6	4.67	4	4	4	4	3	4	4	3.67	4	6	6	5.33
RT	left ear	3	2	3	2.67	2	2	2	2	2	2	2	2	3	2	2	2.33
	right ear	4	4	3	3.67	3	3	2	2.67	2	2	3	2.33	2	1	2	1.67
	left eye	4	4	3	3.67	2	3	4	3	2	2	2	2	2	2	1	1.67
	right eye	3	4	4	3.67	3	3	2	2.67	3	2	2	2.33	3	2	2	2.33
	left front paw	2	2	4	2.67	4	3	3	3.33	2	3	2	2.33	2	2	3	2.33
	right front paw	2	2	4	2.67	3	2	3	2.67	2	2	2	2	1	2	2	1.67
RT+PND	left ear	4	4	6	4.67	4	3	4	3.67	3	3	4	3.33	4	6	4	4.67
	right ear	3	4	4	3.67	4	4	4	4	4	4	6	4.67	4	4	4	4
	left eye	4	3	4	3.67	4	3	4	3.67	6	4	4	4.67	6	6	4	5.33
	right eye	6	4	6	5.33	6	4	6	5.33	4	6	4	4.67	4	6	6	5.33
	left front paw	4	6	6	5.33	4	6	4	4.67	4	4	3	3.67	6	4	4	4.67
	right front paw	4	3	4	3.67	4	4	3	3.67	4	4	6	4.67	4	3	4	3.67
RT+CTS	left ear	6	4	3	4.33	3	2	3	2.67	2	1	2	1.67	2	2	2	2
	right ear	4	4	3	3.67	4	3	3	3.33	2	3	2	2.33	2	2	3	2.33
	left eye	4	4	4	4	2	2	3	2.33	2	2	3	2.33	2	3	2	2.33
	right eye	3	3	4	3.33	4	3	4	3.67	2	2	2	2	3	2	2	2.33
	left front paw	3	3	4	3.33	3	4	3	3.33	4	2	3	3	2	3	2	2.33
	right front paw	3	3	3	3	3	2	3	2.67	3	3	3	3	2	2	3	2.33

MMP-1																	
Time（month）																	
		1				3				5				8			
Group	Sign	No.1 visual	No.2 visual	No.3 visual	Mean	No.1 visual	No.2 visual	No.3 visual	Mean	No.1 visual	No.2 visual	No.3 visual	Mean	No.1 visual	No.2 visual	No.3 visual	Mean
Normal	left ear	3	3	4	3.33	3	3	3	3	4	4	3	3.67	4	3	3	3.33
	right ear	2	3	3	2.67	4	3	4	3.67	4	4	4	4	3	4	4	3.67
	left eye	3	4	4	3.67	4	4	4	4	3	3	2	2.67	4	4	4	4
	right eye	4	4	4	4	3	4	2	3	4	4	4	4	4	3	3	3.33
	left front paw	4	4	3	3.67	4	3	4	3.67	3	4	4	3.67	4	3	3	3.33
	right front paw	3	3	4	3.33	3	4	4	3.67	4	3	3	3.33	3	3	4	3.33
RT	left ear	3	2	2	2.33	2	3	2	2.33	3	2	2	2.33	2	2	1	1.67
	right ear	2	3	3	2.67	2	2	3	2.33	2	1	2	1.67	2	1	2	1.67
	left eye	2	3	3	2.67	3	3	2	2.67	2	2	2	2	1	1	2	1.33
	right eye	2	3	2	2.33	2	3	2	2.33	2	2	1	1.67	1	2	2	1.67
	left front paw	3	2	3	2.67	2	2	3	2.33	2	2	2	2	2	2	1	1.67
	right front paw	3	3	2	2.67	2	2	2	2	2	3	2	2.33	1	2	2	1.67
RT+PND	left ear	2	2	3	2.33	3	2	3	2.67	2	2	3	2.33	3	3	3	3
	right ear	3	3	3	3	3	3	4	3.33	3	3	4	3.33	4	3	3	3.33
	left eye	3	2	3	2.67	2	3	3	2.67	2	3	3	2.67	3	3	4	3.33
	right eye	3	3	2	2.67	3	2	3	2.67	4	3	3	3.33	2	3	3	2.67
	left front paw	3	2	3	2.67	4	3	3	3.33	3	4	3	3.33	3	4	3	3.33
	right front paw	3	3	3	3	3	3	3	3	3	3	2	2.67	4	3	3	3.33
RT+CTS	left ear	3	2	3	2.67	3	4	3	3.33	3	3	3	3	4	4	3	3.67
	right ear	3	3	3	3	3	3	3	3	4	4	3	3.67	3	3	4	3.33
	left eye	2	3	2	2.33	3	2	3	2.67	3	3	4	3.33	3	4	4	3.67
	right eye	3	2	3	2.67	2	3	3	2.67	4	3	3	3.33	4	4	4	4
	left front paw	3	3	3	3	4	3	3	3.33	3	4	4	3.67	3	4	4	3.67
	right front paw	2	3	2	2.33	3	3	4	3.33	4	4	3	3.67	4	3	4	3.67

IL-6 1month

		OD Values						Concentration						oncentration(averag	SEM	epeat of biolog	P value vs. RT	alue vs. RT+PN
Name	Group	1	2	3	4	5	6	1	2	3	4	5	6					
IL-6	Normal	0.15713	0.14935	0.13670	0.14095	0.14241	0.14151	25.64	22.76	18.08	19.65	20.19	19.86	21.03	1.11	6	<0.01	
	RT	0.20346	0.20416	0.19573	0.19819	0.20062	0.21068	42.78	43.04	39.92	40.83	41.73	45.45	42.29	0.79	6		
	RT+PND	0.19316	0.19376	0.19638	0.18659	0.17254	0.17070	38.97	39.19	40.16	36.54	31.34	30.66	36.14	1.70	6	<0.01	
	RT+CTS	0.19565	0.19078	0.20786	0.19895	0.18930	0.20143	39.89	38.09	44.41	41.11	37.54	42.03	40.51	1.05	6	ns	<0.05

IL-6 3month

		OD Values						Concentration						oncentration(averag	SEM	epeat of biolog	P value vs. RT	alue vs. RT+P
Name	Group	1	2	3	4	5	6	1	2	3	4	5	6					
IL-6	Normal	0.13732	0.14538	0.13857	0.14665	0.13970	0.15076	18.31	21.29	18.77	21.76	19.19	23.28	20.43	0.80	6	<0.01	
	RT	0.24584	0.25597	0.26162	0.24281	0.26376	0.24762	58.46	62.21	64.3	57.34	65.09	59.12	61.09	1.32	6		
	RT+PND	0.20141	0.20843	0.21200	0.19122	0.19224	0.19695	42.02	44.62	45.94	38.25	38.63	40.37	41.64	1.29	6	<0.01	
	RT+CTS	0.23365	0.22259	0.23673	0.24138	0.21797	0.22141	53.95	49.86	55.09	56.81	48.15	49.42	52.21	1.44	6	<0.01	<0.01

IL-6 5month																		
		OD Values						Concentration						oncentration(averag	SEM	epeat of biolog	P value vs. RT	value vs. RT+PN
Name	Group	1	2	3	4	5	6	1	2	3	4	5	6					
IL-6	Normal	0.15019	0.14319	0.13803	0.15578	0.14143	0.14530	23.07	20.48	18.57	25.14	19.83	21.26	21.39	0.97	6	<0.01	
	RT	0.26608	0.28654	0.27659	0.26930	0.28349	0.26708	65.95	73.52	69.84	67.14	72.39	66.32	69.19	1.32	6		
	RT+PND	0.23989	0.23176	0.23857	0.21978	0.21178	0.21857	56.26	53.25	55.77	48.82	45.86	48.37	51.39	1.76	6	<0.01	
	RT+CTS	0.24603	0.24170	0.25127	0.26005	0.22935	0.22857	58.53	56.93	60.47	63.72	52.36	52.07	57.35	1.87	6	<0.01	<0.05

IL-6 8month

		OD Values						Concentration						oncentration(averag	SEM	epeat of biolog	P value vs. RT	alue vs. RT+P
Name	Group	1	2	3	4	5	6	1	2	3	4	5	6					
IL-6	Normal	0.14341	0.14095	0.14284	0.15176	0.14565	0.14059	20.56	19.65	20.35	23.65	21.39	19.52	20.85	0.62	6	<0.01	
	RT	0.27227	0.29084	0.28741	0.27824	0.29816	0.27441	68.24	75.11	73.84	70.45	77.82	69.03	72.42	1.54	6		
	RT+PND	0.25170	0.24341	0.24724	0.23395	0.22978	0.24457	60.63	57.56	58.98	54.06	52.52	57.99	56.96	1.25	6	<0.01	
	RT+CTS	0.25100	0.25005	0.25986	0.27470	0.24124	0.23678	60.37	60.02	63.65	69.14	56.76	55.11	60.84	2.06	6	<0.01	ns

IL-10 1month																		
		OD Values						Concentration						Concentration(average)	SEM	[illegible]epeat of biolog[illegible]	P value vs. RT	P value vs. RT+PND
Name	Group	1	2	3	4	5	6	1	2	3	4	5	6					
IL-10	Normal	0.20227	0.19430	0.19916	0.19197	0.20451	0.19170	42.26	39.31	41.11	38.45	43.09	38.35	40.43	0.82	6	<0.01	
	RT	0.24492	0.23451	0.25486	0.23416	0.22854	0.24648	58.04	54.19	61.72	54.06	51.98	58.62	56.44	1.48	6		
	RT+PND	0.22089	0.22432	0.21186	0.22873	0.19719	0.19802	49.15	50.42	45.81	52.05	40.38	40.69	46.42	2.04	6	<0.01	
	RT+CTS	0.21892	0.23624	0.21573	0.22073	0.24321	0.23189	48.42	54.83	47.24	49.09	57.41	53.22	51.70	1.66	6	<0.05	<0.05

IL-10 3month																			
		OD Values						Concentration						Concentration(average)	SEM	epeat of biolog	P value vs. RT	P value vs. RT+PND
Name	Group	1	2	3	4	5	6	1	2	3	4	5	6					
IL-10	Normal	0.20154	0.19631	0.19957	0.18845	0.19320	0.20367	42.09	40.15	41.36	37.24	39	42.88	40.45	0.85	6	<0.01	
	RT	0.28203	0.25220	0.28575	0.25841	0.25045	0.28138	71.91	60.86	73.29	63.16	60.21	71.67	66.85	2.48	6		
	RT+PND	0.22729	0.24181	0.22014	0.23647	0.20818	0.21193	51.63	57.01	48.98	55.03	44.55	45.94	50.52	2.02	6	<0.01	
	RT+CTS	0.23690	0.25323	0.23930	0.24338	0.26235	0.25185	55.19	61.24	56.08	57.59	64.62	60.73	59.24	1.46	6	ns	<0.05

IL-10 5month

		OD Values						Concentration						Concentration(average)	SEM	epeat of biolog	P value vs. RT	P value vs. RT+PND
Name	Group	1	2	3	4	5	6	1	2	3	4	5	6					
IL-10	Normal	0.20089	0.20205	0.19474	0.19736	0.19798	0.20764	41.85	42.28	39.57	40.54	40.77	44.35	41.56	0.68	6	<0.01	
	RT	0.30648	0.27428	0.31042	0.29140	0.27752	0.29323	80.97	69.04	82.43	75.38	70.24	76.06	75.69	2.22	6		
	RT+PND	0.24886	0.26516	0.24200	0.25050	0.22872	0.24532	59.62	65.66	57.08	60.23	52.16	58.31	58.84	1.80	6	<0.01	
	RT+CTS	0.27447	0.26813	0.25577	0.25558	0.27696	0.27393	69.11	66.76	62.18	62.11	70.03	68.91	66.52	1.45	6	<0.01	<0.01

IL-10 8month																		
		OD Values						Concentration						Concentration(average)	SEM	epeat of biolog	P value vs. RT	P value vs. RT+PND
Name	Group	1	2	3	4	5	6	1	2	3	4	5	6					
IL-10	Normal	0.19274	0.19371	0.20135	0.20071	0.20357	0.19442	38.83	39.19	42.02	41.78	42.84	39.45	40.69	0.70	6	<0.01	
	RT	0.31947	0.29204	0.35051	0.32970	0.29906	0.32640	85.78	75.62	97.28	89.57	78.22	88.35	85.80	3.23	6		
	RT+PND	0.26230	0.28853	0.25776	0.26386	0.24837	0.25803	64.6	74.32	62.92	65.18	59.44	63.02	64.91	2.05	6	<0.01	
	RT+CTS	0.29207	0.29315	0.27196	0.27288	0.29914	0.28494	75.63	76.03	68.18	68.52	78.25	72.99	73.27	1.70	6	<0.01	<0.05

CCL3 IHC																	
Time（month）																	
		1				3				5				8			
Group	Sign	No.1 visual	No.2 visual	No.3 visual	Mean	No.1 visual	No.2 visual	No.3 visual	Mean	No.1 visual	No.2 visual	No.3 visual	Mean	No.1 visual	No.2 visual	No.3 visual	Mean
Normal	left ear	1	1	1	1	0	1	1	0.67	1	1	1	1	1	0	1	0.67
	right ear	1	1	0	0.67	1	1	1	1	1	1	1	1	1	1	1	1
	left eye	1	1	1	1	1	0	1	0.67	0	1	1	0.67	0	1	0	0.33
	right eye	1	1	1	1	0	1	0	0.33	1	1	1	1	1	1	1	1
	left front paw	0	1	1	0.67	1	1	1	1	1	1	1	1	1	1	1	1
	right front paw	1	1	1	1	1	1	1	1	1	1	1	1	1	1	1	1
RT	left ear	3	6	4	4.33	6	4	6	5.33	6	8	9	7.67	12	9	12	11
	right ear	4	3	3	3.33	8	4	4	5.33	8	9	8	8.33	12	12	12	12
	left eye	4	6	4	4.67	6	8	8	7.33	9	9	9	9	9	9	12	10
	right eye	4	4	6	4.67	6	4	8	6	12	9	12	11	12	12	9	11
	left front paw	6	6	4	5.33	8	8	6	7.33	8	6	9	7.67	12	12	12	12
	right front paw	4	3	6	4.33	4	6	6	5.33	12	8	9	9.67	9	12	9	10
RT+PND	left ear	3	2	2	2.33	4	3	4	3.67	8	6	6	6.67	9	8	6	7.67
	right ear	4	4	3	3.67	2	4	4	3.33	4	3	6	4.33	8	9	8	8.33
	left eye	2	3	2	2.33	6	2	4	4	6	8	8	7.33	6	6	8	6.67
	right eye	3	3	4	3.33	3	6	4	4.33	3	4	6	4.33	6	6	9	7
	left front paw	3	4	4	3.67	4	3	6	4.33	6	8	6	6.67	8	8	9	8.33
	right front paw	4	2	2	2.67	6	4	4	4.67	6	4	4	4.67	9	6	6	7
RT+CTS	left ear	3	4	3	3.33	6	4	3	4.33	4	4	6	4.67	8	9	9	8.67
	right ear	2	2	4	2.67	6	3	6	5	6	8	6	6.67	6	4	8	6
	left eye	4	3	3	3.33	4	6	4	4.67	4	6	4	4.67	4	6	6	5.33
	right eye	4	3	3	3.33	4	2	4	3.33	6	6	8	6.67	6	6	4	5.33
	left front paw	3	3	4	3.33	4	3	3	3.33	6	4	6	5.33	8	6	9	7.67
	right front paw	4	3	2	3	6	4	4	4.67	6	4	4	4.67	6	8	8	7.33

CCR1 IHC

Time（month）

		1				3				5				8			
Group	Sign	No.1 visual	No.2 visual	No.3 visual	Mean	No.1 visual	No.2 visual	No.3 visual	Mean	No.1 visual	No.2 visual	No.3 visual	Mean	No.1 visual	No.2 visual	No.3 visual	Mean
Normal	left ear	1	1	0	0.67	0	1	0	0.33	1	0	1	0.67	1	1	1	1
	right ear	1	1	1	1	1	1	1	1	1	0	0	0.33	1	1	0	0.67
	left eye	1	0	1	0.67	0	1	1	0.67	0	1	1	0.67	1	1	1	1
	right eye	0	1	0	0.33	1	1	0	0.67	1	1	1	1	0	1	0	0.33
	left front paw	1	1	1	1	0	1	1	0.67	1	0	1	0.67	1	1	1	1
	right front paw	0	1	1	0.67	1	0	0	0.33	0	1	1	0.67	1	0	1	0.67
RT	left ear	3	2	6	3.67	8	6	6	6.67	9	8	9	8.67	12	12	12	12
	right ear	2	2	4	2.67	6	4	6	5.33	9	9	9	9	12	9	12	11
	left eye	4	2	4	3.33	6	4	8	6	8	8	8	8	8	9	12	9.67
	right eye	4	2	2	2.67	6	4	4	4.67	9	8	9	8.67	12	12	12	12
	left front paw	4	2	4	3.33	8	6	6	6.67	9	12	9	10	12	12	9	11
	right front paw	2	3	6	3.67	4	4	8	5.33	9	9	9	9	12	12	12	12
RT+PND	left ear	3	2	2	2.33	6	4	4	4.67	9	6	8	7.67	9	9	8	8.67
	right ear	4	3	4	3.67	4	6	6	5.33	8	8	8	8	8	8	9	8.33
	left eye	2	2	2	2	4	3	3	3.33	8	6	9	7.67	12	9	9	10
	right eye	2	3	2	2.33	6	4	4	4.67	9	9	8	8.67	9	9	8	8.67
	left front paw	3	2	3	2.67	4	4	6	4.67	8	8	6	7.33	9	8	9	8.67
	right front paw	2	3	2	2.33	4	6	4	4.67	9	8	8	8.33	8	6	9	7.67
RT+CTS	left ear	2	2	3	2.33	4	6	6	5.33	6	6	8	6.67	12	9	9	10
	right ear	2	3	2	2.33	6	4	4	4.67	6	4	8	6	8	6	8	7.33
	left eye	2	3	3	2.67	6	3	4	4.33	8	6	8	7.33	8	8	9	8.33
	right eye	3	3	3	3	6	6	8	6.67	8	8	9	8.33	8	8	8	8
	left front paw	3	2	2	2.33	4	4	6	4.67	8	8	8	8	6	4	6	5.33
	right front paw	3	3	3	3	4	4	6	4.67	8	6	8	7.33	9	12	8	9.67
